# Human pairs show collective benefit in olfactory perception despite individual differences and verbal limits

**DOI:** 10.1016/j.isci.2025.114535

**Published:** 2025-12-24

**Authors:** Mustafa Yavuz, Saman Sayahpour, Bahador Bahrami, Ophelia Deroy

**Affiliations:** 1 Graduate School of Systemic Neurosciences, Ludwig-Maximilians-Universität München, Munich, Germany; 2 Department of General Psychology and Education, Ludwig-Maximilians-Universität München, Munich, Germany; 3 Chair of Philosophy of Mind, Faculty of Philosophy, Philosophy of Science and Religious Studies Ludwig-Maximilians-Universität München, Munich, Germany; 4 Munich Center for Neuroscience, Ludwig-Maximilians-Universität München, Munich, Germany; 5 Institute of Philosophy, School of Advanced Study, University of London, London, UK

**Keywords:** neuroscience, sensory neuroscience

## Abstract

Olfaction is a limit case for human communication. Not only does genetic, personal, and cultural diversity make us smell things differently, but perceptual and neurological factors are meant to make it challenging to verbalize our olfactory experiences, besides the affective value that they have for us. Here, we demonstrate that people still achieve a collective benefit in olfactory tasks when allowed to talk to each other, like the benefits only seen in vision and audition. We measured people’s individual smell abilities using a standard clinical test and brought them back after 4 weeks in pairs with matching abilities, others with different ones. We asked everyone to perform discrimination and identification olfactory tasks again, but this time, after giving their own answers, they had to discuss and agree on a joint answer. As in vision, pairs matched in abilities performed better than the best of the two individuals. This joint benefit in olfactory performance overturns the long-held belief that smell resists verbal scaffolding.

## Highlights

• Human pairs exceed the average individual in olfactory perceptual performance
• Collective benefit scales with the pair’s similarity in perceptual ability
• Pairs successfully resolve perceptual conflicts by sharing verbal confidence
• Extensive verbalization impairs odor identification, reflecting lexical limits

## Graphical abstract

**Figure:**
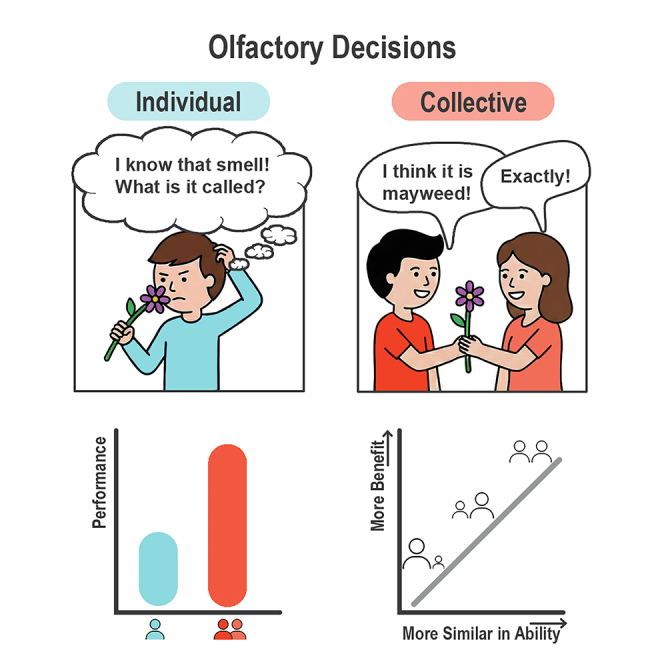

## Introduction

Joint performance in perceptual tasks can exceed what individuals achieve alone, a phenomenon known as the collective benefit. In vision, this effect is well established and depends on two key ingredients: the ability to verbalize one’s perceptual experience and to communicate levels of uncertainty.^1^ By contrast, olfaction is widely believed to lack both. Smell is considered hard to describe, idiosyncratic, and poorly suited for verbal communication.^2^^,^^3^ From this view, letting people talk about what they smell should do little, if anything, to improve their accuracy. This belief shapes both practice and research: whether evaluating wine, fragrance, or food, experts are typically asked to make independent judgments, not to deliberate. Similarly, scientific studies treat smell as something to be measured individually, one nose at a time. It is precisely this orthodoxy we set out to challenge.

Olfactory research done on individuals has certainly brought important insights. Contrary to a 19th century myth,^4^ probably starting with Paul Broca, humans are not so bad at detecting, discriminating and identifying smells. There is compelling evidence for the acuity of the human olfactory system: some compounds can be detected at concentrations as low as 10 parts per billion^5^^,^^6^ and humans are capable of distinguishing between enantiomers—molecules identical in composition but differing in spatial configuration.^7^ Estimates of the total number of odorants humans can discriminate have varied widely from around 10,000 odorants, a more recent and provocative estimate proposed that humans might distinguish up to one trillion.^8^^,^^9^^,^^10^^,^^11^ However, what most individuals in Western populations struggle with is to verbally communicate about odors, even though several non-Western languages exhibit rich, abstract vocabularies for smell.^12^^,^^13^ Common smells, like cinnamon or coffee, can be hard to name; meaning that it is difficult to refer them to their typical sources. If two people disagree on the identity of a smell—one saying it is coffee, the other chocolate—there is little to go on linguistically with. Describing a smell usually elicits generic or metaphorical descriptions, references to autobiographical memories, evaluative expressions (“spicy,” “hot,” “like that gum Big Red”) but not precise abstract characteristics.^13^^,^^14^ This is not only based on observation. Studies consistently show that people are less consistent when naming odors than when naming visual, auditory, or tactile stimuli^2^^,^^3^ and neurocognitive models have proposed stating that olfaction bypasses the pathways that typically support verbal representation for other sensory domains.^15^ In Western cultures especially, olfaction is then seen as a “mute sense”^16^: elusive, subjective, largely isolated from language and therefore impervious to social interactions. But this conclusion faces two challenges. First, the difficulty may not stem from the sensory modality itself, but from a culturally shaped lack of confidence in one’s ability to name and describe smells.^13^^,^^17^ People may hesitate to verbalize olfactory experiences in a test setting not because they cannot discriminate or recognize them, but because they do not want to express responses that they are uncertain of. Second, even if olfactory language differs from say, visual feature-based language, it may still serve its core purpose: enabling effective communication and coordination in a specific context.

Recent research, notably by Asifa Majid and her colleagues, has already transformed the Western-centric view and shown that cultures where coordination around smells plays an important role show a significantly richer set of olfactory verbal strategies.^18^^,^^19^ Languages such as Jahai, Lao, or Umpila feature rich, abstract vocabularies for smell—and users of these languages perform as well naming odors as they do naming colors.^12^^,^^19^ Similarly, in shared contexts, where speakers are both perceptually exposed to the same, limited number of smells, and share some priors on the likelihood of some smells or shared histories, it is possible that verbal strategies used by Western speakers are also doing well.^20^ Existing studies also show that, once provided with background information, individuals can converge better on their verbal descriptions.^21^

Crucially, the idea that olfactory language is poor has rarely been tested in fully interactive settings, where people actively try to communicate and collaborate through smell. Can olfactory communication succeed and let people achieve a collective benefit? This is the question we address by placing smell in paradigms of social calibration used in vision.^1^

Research on collective perceptual decision-making has shown that whether “two heads are better than one” is far from guaranteed—it critically depends on features of the task at hand and the interpersonal dynamics.^22^^,^^23^^,^^24^ A growing body of work has delineated the conditions under which collectives can outperform even the more accurate of their individual members.^22^^,^^25^^,^^26^ In vision, for instance, collaborative benefit emerges when individuals have similar levels of sensory sensitivity, allowing them to efficiently and meaningfully integrate their noisy judgments to achieve higher collective sensitivity.^1^ This principle has been extended beyond vision to domains such as numerical cognition^27^ and even medical image interpretation,^25^^,^^28^ demonstrating that the collective benefit can be achieved but is contingent. Previous works suggest that performance in joint sensory tasks is not solely a function of individual competence, but of similarity of competence, direct verbal communication,^1^^,^^29^ mutual calibration,^22^ and reasonable trust in the shared evidence^30^^,^^31^—a perspective we extend here to olfactory communication.

Some frictions and challenges for fitting olfaction into such sensory collaboration paradigms, some conceptual and some methodological, still need to be addressed upfront. Despite advances in AI-based models predicting perceptual outcomes from molecular structure for single odorants, olfactory perception remains strikingly individual. Inter-rater agreement is consistently found to be low, from older to more recent techniques.^32^^,^^33^ Even in the domain of odor pleasantness, often considered the most primary and universal dimension of olfactory experience,^34^ individual variability persists. Molecular identity explains approximately 41% of the variance in pleasantness ratings, but individual differences still account for 54%, with culture contributing just 6%.^35^ These individual differences are shaped by factors ranging from genetic variation in olfactory receptors,^36^ to personal experience, affective associations, and general attitudes toward smell.^37^

The chemical and neural basis of smell reinforces this individuality. Unlike color vision, where most individuals share the same three photoreceptors, enabling straightforward perceptual matching and from there conceptual and linguistic matching, olfaction involves hundreds of receptor types and millions of potential odorant-receptor interactions.^20^ If each person expresses a unique subset of functional receptors or plasticity, this would make one’s perceptual “odor palette” fundamentally distinct, highlighting the perhaps idiosyncratic and perhaps ineffable nature of olfactory experience. It is therefore important to get good grips on individual differences, and how they are matched, at least at abilities levels, when testing olfaction in a social context.

From a methodological perspective, olfactory psychophysics is much less often done in cognitive science laboratories, even those looking at cross-sensory comparisons. We have to recognize that doing olfactory psychophysics properly is much harder in difficulty because olfaction does not work like vision or audition where well-known engineering principles of signal decomposition (e.g., Fourier transformation into spatial and temporal frequency) have long been recognized to code perceptual features such as contrast, orientation, motion, frequency, or intensity.

Unlike visual or auditory stimuli, repeated exposure to similar smells leads to rapid adaptation or habituation, and odors can linger in the environment, compromising stimulus control.^38^^,^^39^ To work around these issues, we draw on standardized methods from clinical olfactory testing, using localized delivery systems such as odor pens or encapsulated scents.^40^ These recent tools allow for precise, repeatable presentation of odors while minimizing cross-trial contamination—enabling us to systematically evaluate olfactory performance in a socially shared context.

Last but not least, in other sensory domains, especially vision, collaborative performance has been shown to outperform individual judgments when partners can share confidence as a basis to resolve disagreements.^1^^,^^41^ So far, findings on subjective confidence in olfactory performance, or olfactory metacognition for short, have been mixed and fragmented.^42^ Some studies suggest that people have poor access to the accuracy of their olfactory judgments, showing weak correlations between confidence and performance in tasks such as odor identification.^17^^,^^43^ Others, however, report that individuals can track the quality of their perceptual decisions in more constrained discrimination tasks,^44^ indicating that olfactory metacognition may be more context-sensitive than previously assumed. The divergence in results likely stems from differences in task demands (identification vs. discrimination), stimulus familiarity, and how confidence is measured. Importantly, confidence in olfaction also appears to be influenced by general attitudes toward the sense of smell, rather than by moment-to-moment sensory precision alone.^45^ These findings call for a more systematic examination of how metacognitive insight operates in olfaction and how it might be expressed and calibrated in social communicative contexts.

**Figure 1:**
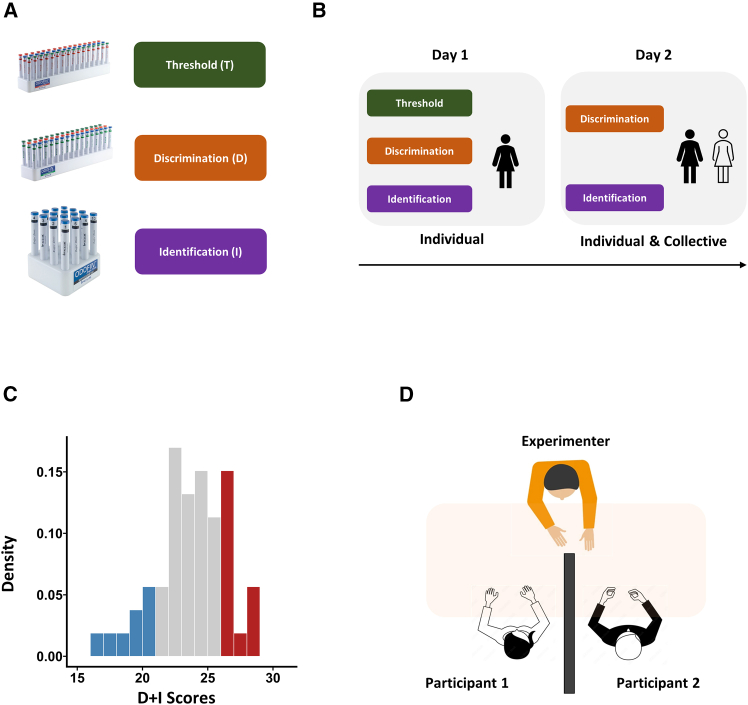
Experimental procedure and setup (A) The Sniffin’ Sticks test battery comprises three olfactory subtests: threshold (T), discrimination (D), and identification (I). (B) On day 1, participants completed all subtests individually. On day 2, only discrimination and identification were repeated, both individually and collectively. (C) Distribution of summed discrimination and identification (D + I) scores across participants. Individuals were paired into dyads based on these scores: participants randomly sampled from the two opposite ends of the distribution (blue and red) were paired to form low similarity dyads; conversely, those with midrange scores (light gray) were randomly paired with each other to form high similarity dyads. (D) Lab setup during dyadic testing on day 2: participants sat side-by-side with a divider between them and across from the experimenter. They were blindfolded for the discrimination test. In each trial, the stimulus pen(s) was handed to them by the experimenter, and then they gave their individual responses silently before engaging in a subsequent timed verbal discussion to reach a joint answer.

Taking these methodological constraints into account, we adapted standardized clinical olfactory tests to a social communicative paradigm—similar to those previously used in vision^1^—to evaluate whether verbal communication about smells is as ineffective as commonly assumed (Figure 1). If olfactory verbalization is fundamentally unreliable and amplifies individual idiosyncrasies, then allowing participants to discuss their judgments should not enhance performance. If, however, individuals can align their olfactory judgments through conversation—even in the absence of a shared, precise vocabulary—this would suggest a previously underestimated capacity for social calibration in smell. Our results support the latter: we find that as long as dyad members have similar levels of perceptual sensitivity, they consistently outperform individuals. Thus, we provide the first empirical evidence that when smelling together, two similar heads are better than one.

## Results

### Demographics and olfactory ability

First, we examined whether age and gender have effects on olfactory ability. For this, we run linear regression models on scores from each of the sub-tests while including age and gender as predictors in separate models. While in this sample age was not a significant predictor for threshold and discrimination scores (threshold: *B* = 0.16, SE = 0.09, 95% CI [–0.03, 0.35], *t*(51) = 1.74, *p* = 0.088; discrimination: *B* = 0.02, SE = 0.07, 95% CI [–0.12, 0.15], *t*(51) = 0.23, *p* = 0.818), it was significant for identification, *B* = 0.16, SE = 0.06, 95% CI [0.05, 0.27], *t*(51) = 2.91, *p* = 0.005. On the other hand, gender did not significantly predict performance in any subtest (threshold: *B* = −0.09, SE = 0.84, 95% CI [–1.78, 1.60], *t*(51) = –0.11, *p* = 0.913; discrimination: *B* = −0.59, SE = 0.59, 95% CI [–1.77, 0.60], *t*(51) = –0.99, *p* = 0.323; identification: *B* = −0.26, SE = 0.53, 95% CI [–1.32, 0.79], *t*(51) = –0.50, *p* = 0.616).

### Are people metacognitively sensitive to their olfactory performance?

**Figure 2:**
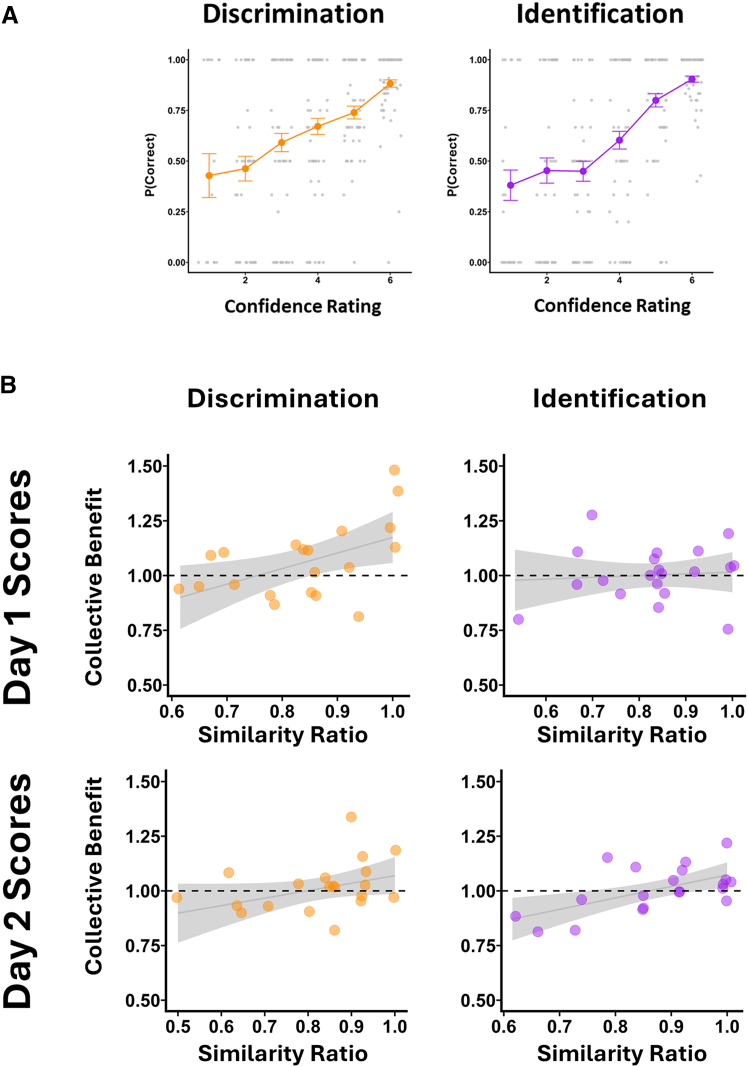
Accuracy—confidence and collective benefit—similarity ratio relationships across task types (A) Accuracy across confidence ratings for discrimination and identification. Orange and purple lines show the probability of correct values across each confidence rating level, averaged across participants. Each gray dot is one subject average. Error bars: ±1 SEM. (B) Relationship between similarity ratios of individuals and collective benefit. Relationship between dyad members’ perceptual similarity and collective benefit. Top row: collective benefit as a function of similarity ratio on day 1, separately for performance in the discrimination task (left, orange) and the identification task (right, purple). Bottom row: the same relationship on day 2. Each dot represents one dyad; shaded regions show 95% confidence intervals around the regression line. The horizontal dashed lines at *y* = 1 indicate the threshold for collective benefit.

Given the controversy among previous empirical investigations of olfactory metacognition (see “introduction”), we set out to establish if our participant’s data obtained in the isolated condition could provide any evidence to support the existence of olfactory metacognition. To this end, following standard practice in assessment of metacognition,^46^ we computed the proportion of correct responses at each level of confidence ratings. Our mixed-effect logistic regression models showed that for both discrimination (OR = 1.69, 95% CI [1.49, 1.93], *z* = 8.01, *p* < 0.001) and identification (OR = 2.00, 95% CI [1.72, 2.33], *z* = 9.03, *p* < 0.001) participants’ confidence ratings were strongly correlated with the accuracy of their choices (Figure 2A). This provides strong evidence for demonstration of olfactory metacognition, needless to say, within the empirical framework defined by the Sniffin’ Sticks test. Note that without such evidence, it would be much harder to justify or even envisage the possibility of any form of social communicative collective perceptual olfactory judgment to take place.^22^

### Are collectives better than individuals?

Having produced evidence for olfactory metacognition in private decisions, we then moved on to compare individual and collective performance. We used two approaches. As a reminder, the collaborative score corresponds to the answers agreed upon by the two participants after discussion. First, as pre-registered, we examined if, across the board and independent of any contextual condition, two heads would be better than one in collective olfaction. For each dyad, we compared the better individual’s score to the collaborative score for each subtest. Consistent with previous findings,^22^^,^^23^ paired *t* test results showed that neither for discrimination (*t*(19) = −1.29, *p* = 0.212, 95% CI [−1.44, 0.34]) nor for identification (*t*(19) = 0.00, *p* = 1.00, 95% CI [−0.70, 0.70]) there were no significant overall differences between collaborative and better individual’s performances.

As a second exploratory analysis, we compared the collaborative performance to the average of individual scores from the dyad. Dyad scores were significantly higher than average of the individuals for both discrimination (*t*(19) = −4.53, *p* < 0.001, 95% CI [−2.38, −0.87]) and identification (*t*(19) = −3.10, *p* = 0.006, 95% CI [−1.84, −0.36]) tests. Already this finding shows us that, when individuals collaborate in joint olfaction, they must be doing more than flipping a coin between them to resolve disagreements. This average is what one would expect from a simple “voting” model of social interactive decision-making.^1^ Communication, this result shows, enables people to rise above this lower benchmark set by simple voting as the simplest mechanism of joint decision-making.

### Does the collective benefit vary with ability similarity?

We then moved on to test our second pre-registered hypothesis (Figure 2B). We focused here on the collective benefit, defined as the ratio between the dyad’s score and the test score of the better performer of that dyad. Scores above 1.00 indicate that performing the task in a dyad outperforms its best individual. Crucially, the term collective benefit here differs from the collaborative performance in the previous section. While the collaborative performance is simply the raw score of the joint decisions made by the pair, collective benefit captures the extent to which joint performance exceeds a given team’s best individual capacity.

Similarity ratio between the individuals was defined as the ratio *S*_*min*_*/S*_*max*_ by where *S* corresponds to a given individual’s score in the test of interest (e.g., identification or discrimination) and *max* and *min* indicate—within the dyad—the lower and the higher score; similarity ratio was computed either from day 1 or from day 2 individual scores, as specified. If the two individuals’ scores are very similar to each other (*S*_*max*_ ≃ *S*_*min*_), this ratio would be close to 1; conversely, if one dyad member is far better than the other (i.e., *S*_*max*_ *>> S*_*min*_) then the ratio is close to zero. Note that, because our participants made individual judgements on both days, we could calculate the *S*_*max*_ and *S*_*min*_ based on their individual performances on either day 1 or day 2. Because joint decisions were only elicited on day 2, any relationship between similarity ratio drawn from day 1 data and collective benefit from day 2 (i.e., some 40 days later) would constitute strong evidence for predictability of collective benefit. Note that “Similar” and “Different” dyads did not differ in mean individual ability scores on either test (Welch tests, both ns; see supplemental information).

For the discrimination task, the similarity ratio was a significant predictor of collective benefit using similarity from day 1 (*B* = 0.71, SE = 0.27, *t*(18) = 2.60, *p* = 0.018, 95% CI [0.13, 1.29]) and marginally significant for calculating similarity for day 2 (*B* = 0.34, *t*(18) = 1.87, *p* = 0.078, 95% CI [−0.04, 0.73]). For the identification performance, the similarity ratio from day 2 was a significant predictor of collective benefit, *B* = 0.52, *t*(18) = 3.22, *p* = 0.005, 95% CI [0.18, 0.87], while the similarity ratio from day 1 was not (*B* = 0.08, SE = 0.21, *t*(18) = 0.38, *p* = 0.71, 95% CI [–0.36, 0.52]).

### Does ability similarity help solve conflicts?

Having demonstrated the relevance of similarity of ability for collective benefit, we then moved on to examine the possible mechanisms through which similarity may determine collective success. We first focused on whether there is a relationship between similarity and the number of conflicts experienced within a dyad and their success in resolving it. Conflict trials, for each task, were defined as when there is a mismatch between the answers of individuals in a given trial. There was, on average, similar number (*t*(19) = 0.40, *p* = 0.695) of conflict trials in the discrimination (*M* = 5.45, *SD* = 2.21, 34% of total trials) and identification (*M* = 5.20, *SD* = 1.74, 32.5% of total trials) tests. A conflict is considered to be successfully resolved when the collective response was correct. Since conflicts in our design can arise either when exactly one partner is correct or when both partners are wrong (18.7% of discrimination conflicts and 22.4% of identification conflicts); analyses that focused on conflict trials were restricted to the former case (one correct and one incorrect). For context, trials where both partners were initially correct almost always remained correct after discussion (100% for both Discrimination and Identification), whereas trials where both partners were initially wrong rarely recovered (4% in discrimination; 0% in identification); further details of these pre-discussion states and post-discussion outcomes are reported in the supplemental (Tables S45 and S46). Dyads were similarly successful in resolving their conflict in the discrimination (*M* = 72% *SD* = 20%) and in the identification (*M* = 64% *SD* = 21%), with no significant difference between the two tests (*t*(19) = 1.64, *p* = 0.118).

**Figure 3:**
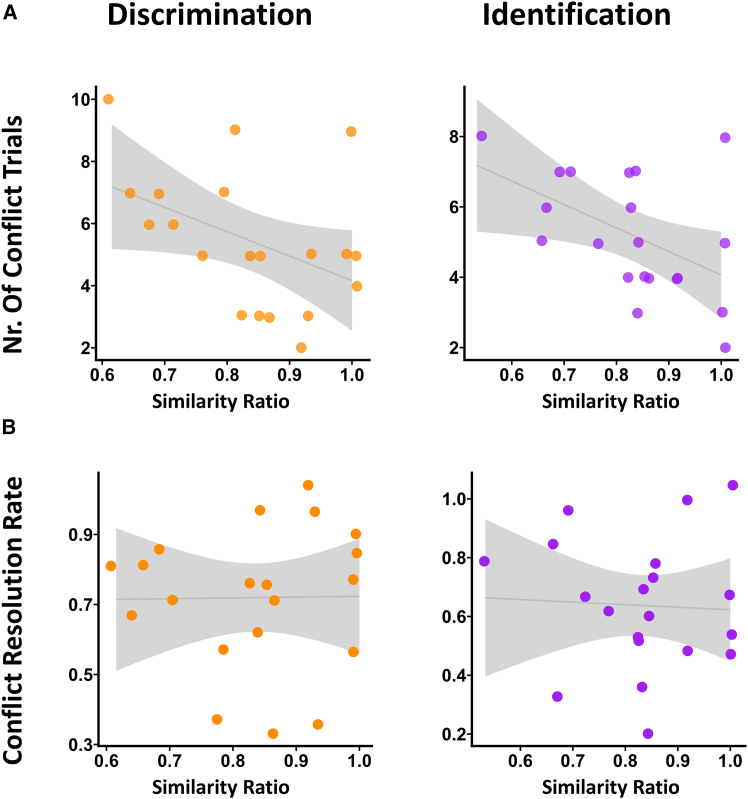
Ability similarity, number of conflict trials and conflict resolution Relationships between dyad members’ perceptual similarity and number of conflicts experienced (A) and conflict resolution rates (B), separately for performance in the discrimination task (left, orange) and the identification task (right, purple). Each dot represents one dyad; shaded regions show 95% confidence intervals around the regression line.

The similarity ratio (drawn from day 1 scores) predicted the number of conflict trials (Figure 3A). Our linear regression models showed that there was a negative and marginally significant relationship between similarity ratio and number of conflicts in discrimination (*B* = −7.84, SE = 3.79, *t*(18) = −2.07, *p* = 0.053, 95% CI [–15.81, 0.12]), while the negative association was significant for identification, *B* = −6.67, SE = 2.78, *t*(18) = −2.40, *p* = 0.028, 95% CI [–12.52, −0.83]. However, similarity ratio did not correlate with successful resolution of conflict (Figure 3B). Regression models yielded non-significant results for both discrimination (*B* = 0.02, SE = 0.39, *t*(18) = 0.06, *p* = 0.953, 95% CI [–0.79, 0.83]) and identification (*B* = −0.09, SE = 0.40, *t*(18) = −0.22, *p* = 0.827, 95% CI [–0.92, 0.74]).

### Verbal communication of olfactory judgments

Given the dominant idea that people struggle to communicate about smell, the evidence of a collective benefit in matched pairs represents a significant finding, but also an opportunity to address a new question: What supports successful communication and conflict resolution about smells? To answer this question, we turned to the voice recordings of our participants while they make collective decisions. We first computed 3 metrics based on audio transcriptions: the number of words uttered during a trial by each participant, the total number of words uttered by the dyad, and finally, the number of certainty, uncertainty, and confidence sharing words and phrases. Because trial-level utterances were short, we focused on word counts and the confidence-lexicon summary; richer content analysis (e.g., polarity or discourse functions) was not attempted.

**Figure 4:**
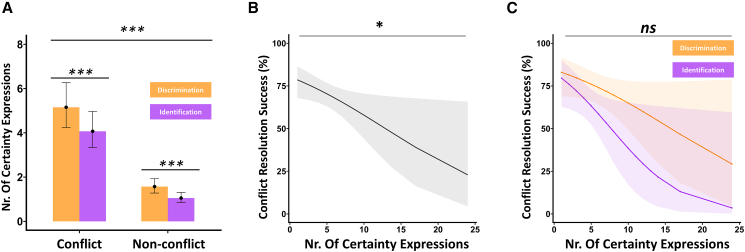
Impact of certainty expressions on conflict resolution success in dyadic olfactory decision-making (A) Mean number of certainty-related expressions uttered per dyad during conflict and non-conflict trials, separately for discrimination (orange) and identification (purple) tasks. (B) Model-predicted probability of successful conflict resolution as a function of the number of certainty expressions, collapsed across task types. (C) The same model as in (B), plotted separately for the discrimination (orange) and identification (purple) tasks. Shaded regions represent 95% predicted confidence intervals. Error bars in (A) represent ±1 SEM. ∗∗∗*p* < 0.001, ∗*p* < 0.05, ns, non-significant.

**Figure 5:**
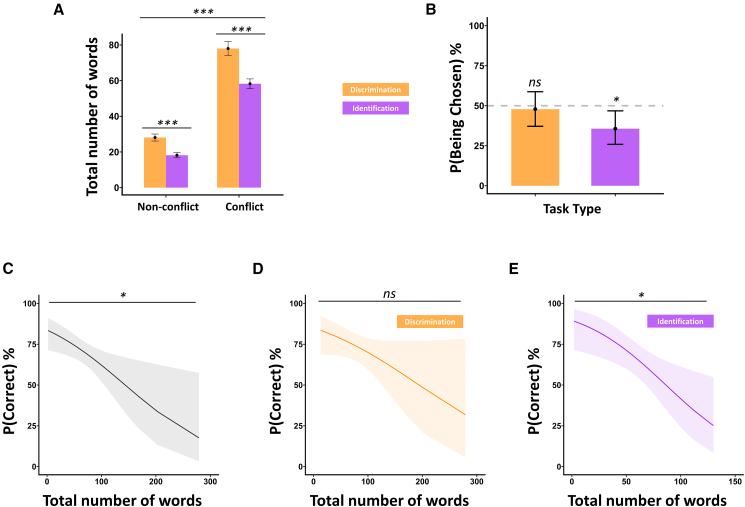
Verbal communication, task type, and conflict resolution in dyadic olfactory decision-making (A) Mean total number of words uttered per dyad during non-conflict and conflict trials, separately for discrimination (orange) and identification (purple) tasks. (B) Probability that the more talkative individual’s initial answer was chosen as the dyad’s joint decision, plotted separately by task type. (C) Model-predicted probability of successful conflict resolution (i.e., dyad giving the correct answer in conflict trials) as a function of the total number of words spoken, across both task types. (D and E) The same model as in (C), plotted separately for the discrimination (D, orange) and identification (E, purple) tasks. Shaded regions represent 95% predicted confidence intervals. Error bars in (A) and (B) represent ±1 SEM. ∗∗∗*p* < 0.001, ∗*p* < 0.05, ns, non-significant.

As one would expect, people talked more when they disagreed. The linear mixed-effects model showed a main effect of trial type (conflict vs. non-conflict) (Figures 4A and 4B = 47.90, SE = 2.93, 95% CI [42.15, 53.65], *t*(617) = 16.35, *p* < 0.001). More interestingly, task type (discrimination vs. identification) was also a significant predictor of the total number of words uttered in a trial. People talked more in the discrimination trials than identification trials (Figures 5A and 5B = −11.47, SE = 2.38, 95% CI [–16.13, −6.80], *t*(617) = −4.83, *p* < 0.001). The interaction was not significant.

After these initial sanity checks, we focused on what could predict success in conflict resolution. To this end, we filtered the dataset and solely focused on conflict trials. The logistic mixed-effects model showed a significant main effect of the total number of words, talking less produced better conflict resolution (OR = 0.99, SE = 0.004, *z* = −2.55, *p* = 0.01, 95% CI [0.98, 1.00]). Next, we focused on whether this effect is task specific, given our previously shown findings. In the identification task, the total number of words exchanged in a trial was a significant predictor of conflict resolution success: participants used fewer words during successfully resolved conflicts (OR = 0.98, SE = 0.09, *z* = −2.72, *p* = 0.01 [corrected], 95% CI [0.96, 0.99]). In the discrimination task, however, we did not find a similar relationship (OR = 0.99, SE = 0.005, *z* = −1.73, *p* = 0.17 [corrected], 95% CI [0.98, 1.00]) (Figures 5C–5E).

Following these findings, to determine whether this pattern reflects stable talkativeness (at dyadic or individual level) versus trial-by-trial verbosity, we computed three word-use components on identification conflict trials: a within-dyad deviation (words uttered in a trial minus that dyad’s mean number of words uttered across Identification conflicts), a between-dyad component (each dyad’s mean talkativeness, centered on the grand mean), and an individual-level talkativeness-imbalance index within each dyad (|Mean Person 1 − Mean Person 2|/(Mean Person 1 + Mean Person 2)). A mixed-effects logistic regression predicting dyadic accuracy from these three components (fixed effects), with a random intercept for dyad, showed that talking more than a dyad’s own average on a given conflict trial predicted lower success (within: *b* = −0.04, SE = 0.01, *z* = −3.58, 95% CI [−0.07, −0.02], *p* < 0.001), whereas between-dyad talkativeness was not significant (between: *b* = −0.01, SE = 0.01, *z* = −0.62, 95% CI [−0.07, 0.02], *p* = 0.53) and the individual-level talkativeness imbalance was also not significant (*b* = −1.90, SE = 2.26, *z* = −0.84, 95% CI [−6.33, 2.53], *p* = 0.40). The within-dyad effect remained significant when including the total confidence-lexicon count as a covariate (within: *b* = −0.05, SE = 0.02, *z* = −3.23, 95% CI [−0.08, −0.02], *p* = 0.001). Together, these results suggest that the identification effect reflects trial-specific verbosity rather than general talkativeness at the dyad or individual level.

Finally, given that more communication in identification tasks is associated with lower success levels in conflict trials, we could ask whose decision is selected as the collective response at the end. On a trial-by-trial basis, we classified the “more” and “less” talkative individuals within each dyad, based on the number of words uttered during the identification task. We then tested whether the more talkative individual was more likely to have their initial choice selected as the collective response. A mixed-effects logistic regression model with a random intercept for dyad revealed that the more talkative individual was significantly *less* likely to win the conflict (*B* = −0.59, SE = 0.24, *z* = −2.50, *p* = 0.012). This corresponds to a probability of only 35.7% (95% CI [25.9%–46.6%]) that the more talkative individual’s decision was chosen (Figure 5B).

### Content of verbal communication, task type, and conflict resolution

Finally, we examined whether the verbal shared content between individuals contributes to collective decision-making. Similar to the previous section, we begin with an important sanity check. A generalized linear mixed model with a Poisson distribution was used to predict conflict resolution success based on counts of certainty expression. There was a significant main effect of conflict, such that dyads produced fewer confidence words in the non-conflict trials compared to the conflict trials, *b* = 1.26, SE = 0.07, *z* = 16.97, 95% CI [1.12 1.41], *p* < 0.001. There was also a significant main effect of task type, with fewer confidence-related words in the identification trials, *b* = −0.35, SE = 0.09, *z* = −3.82, 95% CI [–0.53–0.17], *p* < 0.001. The interaction between conflict and trial type was not statistically significant, *b* = 0.14, SE = 0.11, *z* = 1.25, 95% CI [–0.08 0.36], *p* = 0.21 (Figure 4A).

We then focused on whether exchange of confidence and uncertainty influences success at conflict resolution. For this, we only focused on conflict trials. A mixed-effects logistic regression revealed a significant effect of expressed verbal confidence on dyadic decision accuracy. Higher total confidence word count was negatively related to successful conflict resolution (OR = 0.85, SE = 0.06, *z* = −2.66, *p* = 0.01, 95% CI [0.76, 0.96]). In a second model, we included task type (discrimination vs. identification) and its interaction with expressed confidence as additional predictors. This time, the main effect of confidence words was marginally significant (OR = 0.882, SE = 0.07, *z* = −1.73, *p* = 0.08, 95% CI [0.765, 1.016]), and neither the main effect of task type (OR = 1.50, SE = 0.74, *z* = 0.55, *p* = 0.58, 95% CI [0.35, 6.33]) nor the interaction (OR = 0.81, SE = 0.13, *z* = −1.61, *p* = 0.11, 95% CI [0.623, 1.047]) reached significance (Figures 4B and 4C). Lastly, a Bayesian model comparison was conducted to evaluate whether including an interaction between trial type and the number of confidence words improved model fit. The estimated Bayes factor comparing the simple model to the complex model was BF_01_ = 1.70, providing anecdotal evidence in favor of the simpler model (i.e., against including the interaction^47^).

## Discussion

In this study, we asked whether people can achieve collective benefits when making olfactory judgments together—despite the well-known challenges of communicating about smells due to well-documented psychological and biological barriers. Using a set of standardized clinical smell tests, participants first completed discrimination and identification tasks individually. After a delay of several weeks, they returned in pairs of matched or mismatched ability (as planned by design) to repeat the tasks. Crucially, they were required to first decide privately, then reach a joint decision through verbal discussion. This design allowed us to (1) test whether collaborative olfactory performance can exceed individual performance, (2) whether interpersonal ability similarity predicts the emergence of such a collective benefit, and (3) for the first time, to identify the quantitative profile of olfactory communication during joint decisions using state-of-the-art language processing tools.

Our results show clear evidence for olfactory metacognition: participants’ confidence ratings reliably predicted the accuracy of their odor judgments. This matters because previous studies have reported mixed findings on whether people have metacognitive access to olfaction. We suggest that our success stems from using the Sniffin’ Sticks test, a standardized tool that allows precise, forced-choice olfactory decisions. Its structure mirrors established psychophysical methods in vision and audition, offering a reliable way to measure both performance and confidence. This framework may help resolve past inconsistencies and clarify the conditions under which olfactory metacognition can be detected.

Moreover, we found that dyadic performance reliably exceeded the average performance of the individuals within each dyad. This result is important because it rules out a simple but influential model of collective decision-making: the voting model,^48^^,^^49^^,^^50^ in which joint responses are assumed to reflect either agreement or random resolution of disagreement. If dyads had merely voted and flipped a coin in cases of conflict, their collective performance would have matched the average of their individual scores. Instead, the observed improvement suggests that communication contributed meaningfully to performance, indicating that dyads integrated information rather than relying on chance or minimal coordination. Our findings therefore align with research on deliberative benefits in group interactions, where discussion drives better decisions.^1^^,^^51^^,^^52^^,^^53^

A central finding of our study is that collective benefit—how much better a dyad performs compared to its best individual member—is predicted by the similarity in sensory sensitivity between the two individuals. Importantly, this predictive relationship held despite some ∼40-day gap between sessions: ability similarity was measured on day 1, while joint performance was assessed on day 2. To rule out a mean-ability confound, we verified that “Similar” and “Different” dyads did not differ in mean individual scores for discrimination or identification. This result is crucial for several reasons. First, it replicates and extends prior findings from vision,^1^ numerical cognition,^27^ and medical image classification.^25^^,^^28^ Second, it offers a practical framework for assembling teams—such as basketball referees, medical diagnosis panels, etc.—according to similarity in ability in order to optimize group outcomes. Third, the link between ability similarity and collective benefit originates from a previous model of interactive decision-making,^1^ where disagreements are resolved through comparing individual confidence levels. Our findings suggest that, at least in the discrimination task employed here, participants likely resolved conflicts by exchanging and weighing confidence in their initial judgments.

To better understand how people communicate about smells in joint decision-making, we recorded participants’ verbal interactions during the olfactory tasks and used state-of-the-art language processing tools to transcribe and analyze these conversations. To our knowledge, this is the first study to quantitatively examine the verbal dynamics of olfactory collaboration. Given this gap in the literature, we first established the reliability of our approach through key sanity checks—for example, showing that people spoke more when they disagreed. Most strikingly, we found that discussions were longest during the most difficult trials—those where dyad members initially disagreed and ultimately reached an incorrect joint decision. This provides strong, implicit evidence for olfactory metacognition, critically extracted from naturally embedded communication. This result goes beyond standard laboratory-based, task-specific measures of metacognition^46^—such as the correlation between confidence and accuracy—and offers clear, compelling evidence for the observation of ecologically valid use of metacognition in social interaction. It shows that people do not merely possess metacognitive insight into their olfactory judgments in isolation, but actively express and deploy that insight when coordinating with others. In the olfactory domain, long considered resistant to reflection and communication, this embedded metacognitive behavior marks a significant shift in how we understand the social potential of smell.

An alternative interpretation of this finding is to argue that producing less words during communication was associated with higher success in resolving individual conflicts. This may reflect the limited olfactory vocabulary in English—an issue compounded by the fact that our participants, though all fluent in English, came from diverse linguistic backgrounds and were instructed to speak in English. The language constraint likely added difficulty to an already lexically impoverished domain. Supporting this interpretation, we observed that participants spoke significantly less during identification than discrimination tasks, perhaps because identification demands more domain-specific vocabulary, which participants lacked or struggled to access.

Our results already challenge the dominant approach in olfactory science, where performance and descriptions are almost exclusively gathered through isolated individual trials. Even in perfume training and clinical olfactory assessment, descriptors are typically developed through repeated individual exposure and memorization, rather than negotiated interaction. But our findings echo a broader trend in collective intelligence research, which shows that interaction—not just averaging independent judgments—produces more accurate and robust decisions.^1^^,^^24^ In this case, better decisions—more accurate odor discrimination and identification—may translate into more informative, calibrated, and context-sensitive descriptions.

This has far-reaching implications. First, it suggests that the perceived limitations of olfactory language may be overstated. In prior work, we have argued that olfactory naming does not depend on feature-based categorization (as in color or shape), but on communicative and metacognitive resources that allow people to align their interpretations across subjective variability.^13^ The fact that dyads can outperform individuals in discrimination and identification supports the view that language for smell is not absent—but flexible, interactional, and task-sensitive.

Importantly, we observed a joint benefit in the absence of feedback, suggesting that social deliberation can bootstrap accuracy in perceptual domains that lack evidence of objective ground truth. Even when participants cannot access the odor source, social deliberation appears to improve perceptual decisions, potentially via candidate mechanisms such as confidence exchange, calibration, and error checking, including the mapping between percepts and labels, rather than altering low-level sensory representations. In this way, our results extend previous work on joint visual detection^1^^,^^24^ into the olfactory domain; where uncertainty is higher, vocabulary is weaker, and perceptual norms are less stable. We also see these results in the absence of information on the collaborator’s reliability.

These findings open new avenues for olfactory science. Rather than relying solely on individual descriptions to create olfactory taxonomies or train artificial noses and translate chemicals into verbal classifiers, future work could incorporate interactive tasks that tap into social calibration. This would better reflect how people actually use smell in daily life, not in isolation, but embedded in social rituals, cooking, intimacy, or care. It also points toward new tools for fields like perfumery and wine tasting, where more accurate decisions and communicatively robust descriptors could emerge from pair or group deliberation, not just rote individual learning. Finally, this work invites a conceptual shift. If olfactory language is co-constructed through interaction in context, then its limitations may reflect testing paradigms, not cognitive constraints.^54^ By creating opportunities for people to discuss, negotiate, and align their experiences, we can see that olfaction—like vision—is more social than we thought.

While our work focused on dyads, the same logic could extend to larger or differently composed groups in two ways. First, experts (e.g., perfumers, sensory assessors, and sommeliers) could be an interesting target population, particularly given our finding that more extensive exchange can reduce dyadic accuracy in identification. Experts’ extensive and possibly shared lexicon may enable brief, criterion-focused discussions to calibrate confidence and aggregate judgments. However, since our current battery would likely be too easy for such experts, especially for identification, future studies can use either free recall or multiple-choice response sets with hard, confusable distractors to avoid ceiling and reveal how brief discussion shapes calibration. Second, a wisdom-of-crowds approach can be implemented by increasing group size in general public contexts (e.g., science fairs, and exhibitions). In both cases, especially for larger groups where free discussion might take very long, structured confidence sharing (e.g., providing private numeric confidence which is followed by a brief, standardized exchange) preserves pre-discussion independence and enables rapid, confidence-based dyadic decision.

### Limitations of the study

Our study establishes promising findings, which should be put in perspective. Our primary aim was to test the matching-by-similarity hypothesis across multiple olfactory tasks, which required substantial experimental commitment—over 150 h including re-recruitments. This limited the number of interactions and the duration of each, constraining our ability to explore how communication evolves within dyads over time. Mechanisms such as linguistic alignment, convergence, and strategy formation—shown to predict collective benefit in other domains^24^—need a larger corpus to be assessed. We therefore did not analyze polarity (certainty vs. uncertainty) or discourse functions (e.g., evidence citation, checking questions, and proposals/acceptance), as trial-level exchanges were brief and would not support reliable coding and inter-rater agreement; future studies with longer discussions and targeted annotation schemes can test how these features contribute to successful conflict resolution. Likewise, we did not model fine-grained error patterns (e.g., which odors were more confusing for the participants or which distractors were chosen) because the number of trials per odor/alternative was insufficient for stable estimates; future studies including bigger stimuli sets could test whether complementary error profiles within a dyad predict greater collective benefit.

Similarly, we focused on verbal communication, and it is possible in the future to also analyze non-verbal cues such as prosody, facial expressions, or gestures, which are known to serve as metacognitive signals in joint decision-making.^51^ Future studies incorporating multimodal analyses could shed light on how different channels contribute to confidence sharing and conflict resolution, particularly in a domain like olfaction where language may be less precise.

Another constraint was the use of multiple-choice options in the identification task, which may have restricted the range of responses and limited the emergence of shared descriptive vocabularies. The choice for this format came from the choice to stick with a standardized and well-respected olfactory testing instrument. Using open-ended formats could reveal how people generate and align olfactory concepts in more naturalistic settings.

In our sample, discrimination showed higher test-retest reliability than identification, yet neither subtest exhibited a systematic mean shift between sessions. Nonetheless, transient olfactory changes (e.g., viral infections and allergic rhinitis) may introduce additional noise, likely attenuating observed associations. Future studies, especially in populations with greater variability, should include a brief rapid olfactory screen at each session (or reschedule during acute symptoms) to document day-level ability and reduce reliance on self-reported health status.

Finally, our study was conducted primarily in English with Western participants, many of whom may lack cultural or linguistic familiarity with rich olfactory vocabularies. Relatedly, we did not obtain standardized measures of language proficiency; although all dyads comfortably completed the English discussion, future work should record language proficiency and test for language effects on performance and conversational dynamics. Cross-cultural research, especially in communities where olfactory language is more developed (e.g., Jahai or Umpila speakers), could determine whether collective benefits rely less on sensitivity matching and more on shared linguistic resources. Together, these extensions will help clarify how robust the observed benefits are and deepen our understanding of how olfactory communication and metacognition function in everyday social interactions.

## Resource availability

### Lead contact

Further information and requests for resources and reagents should be directed to and will be fulfilled by the lead contact, Mustafa Yavuz (Yavuz.Mustafa@lmu.de).

### Materials availability

This study did not generate new unique reagents.

### Data and code availability

• All data reported in this paper has been deposited at OSF and is publicly available as of the date of publication. The accession number/DOI is listed in the key resources table.
• All original code has been deposited at OSF and is publicly available as of the date of publication. The DOI is listed in the key resources table.
• Any additional information required to reanalyze the data reported in this paper is available from the lead contact upon request.

## Acknowledgments

O.D. was supported by the 10.13039/501100001663Volkswagen Foundation Grant Co-Sense and the Excellence Funding from the 10.13039/100004807DFG/10.13039/100017678LMU investment funds. B.B. and M.Y. were further supported by the 10.13039/100010663European Research Council (ERC) under the European Union’s Horizon 2020 research and innovation program (819040—acronym: rid-O). We also thank Selenay Yıldırım for her assistance with the design of the graphical abstract.

## Author contributions

M.Y., S.S., B.B., and O.D. conceived the study, designed experiment, interpreted the data; M.Y., B.B., and O.D. supervised the study; M.Y. and S.S. recruited the subjects for olfactory testing; S.S. conducted olfactory testing; M.Y. and S.S. conducted data analysis of behavioral data; M.Y. and S.S. conducted the transcription of voice recordings; M.Y., S.S., B.B., and O.D. interpreted the results; M.Y. coordinated the drafting of the manuscript, submission, and revision processes with the contributions of all co-authors. All authors have read and approved the final version of the manuscript.

## Declaration of interests

The authors declare no competing interests.

## Declaration of generative AI and AI-assisted technologies in the writing process

During the preparation of this work, the authors used ChatGPT (GPT-5.1 Thinking, OpenAI) in order to assist with language editing (spelling, grammar, and syntax). After using this tool, the authors reviewed and edited the content as needed and take full responsibility for the content of the published article.

## STAR★Methods

### Key resources table

**Table:** 

REAGENT or RESOURCE	SOURCE	IDENTIFIER
**Deposited data**		

Analyzed data and scripts	This paper	https://osf.io/s5adx/overview?view_only=22feb396861f4b8c9d3ce0e74892b742

**Software and algorithms**		

Audacity version 3.5.1	Audacity Team. (2014). Audacity(R): Free Audio Editor and Recorder	https://sourceforge.net/projects/audacity/
RStudio version 2025.09.1	Posit team (2025). RStudio: Integrated Development Environment for R. Posit Software, PBC, Boston, MA.	https://posit.co/download/rstudio-desktop/
Restream audio-to-text transcription	2025 Restream, Inc.	https://restream.io/tools/transcribe-audio-to-text

**Other**		

Olfactory Threshold Test n-Butanol	Burghart Messtechnik GmbH	Cat# LA-13-00009
Olfactory Discrimination Test	Burghart Messtechnik GmbH	Cat# LA-13-00011
Olfactory Identification Test 16, blue	Burghart Messtechnik GmbH	Cat# LA-13-00013
Multi-Plattform-Stereo-Headset PC 5 CHAT	EPOS	Cat# 1000445

### Experimental model and study participant details

We recruited 53 healthy adult individual participants (Age: Mean = 24.42, SD = 4.07; 16 Males, 37 Females) who took the Day 1 test. Of these, 40 participants returned for Day 2 (Age: Mean = 24.53, SD = 4.03; 12 Males, 28 Females). Participants provided verbal and written informed consent prior to the study and were compensated for with money. Eligibility included the ability to communicate in English at an everyday conversational level. We did not collect standardized proficiency scores but recorded participants’ self-reported native language status and checked for its effect on the performance and linguistic outcomes, which are reported in the supplementary material (Section G). This study was approved by the Ethics Committee of Faculty 10, LMU-Munich (Nr. 224810). Participants were recruited after passing the following exclusion criteria: (i) any current or recent (<4 weeks) condition that could have impaired olfaction, for example nasal obstruction or sinus disease, acute upper-respiratory infection, chronic cardiovascular, endocrine, autoimmune or pulmonary disorder, recent (<1 year) upper-aerodigestive surgery, or a history of head trauma; (ii) self-reported history of smoking; (iii) history of neuropsychiatric disorder; (iv) pregnancy; and (v) psychophysical evidence of anosmia. These criteria are laid out in our pre-registration at: https://osf.io/gj9wm/?view_only=2965fb5fc2fc40b6b713354eb042749e. Race, ethnicity, and ancestry data were not collected. We analyzed the influence of gender on performance and found no significant effects (see results).

### Method details

#### Battery of olfactory tasks

We used the *Sniffin’ Sticks* test to assess olfactory threshold, discrimination, and identification (Burghart Messtechnik GmbH, Germany). The Sniffin’ Sticks Test is a standardized kit for assessing chemosensory performance by a set of pen-like odor dispensing devices,^55^^,^^56^^,^^57^ validated in both clinical and research settings.^58^ The full kit includes three tests—threshold, discrimination, and identification (T, D, and I) producing a separate score for each and a combined total score (TDI).

#### Threshold test

The threshold test consists of 16 triplets of pens (a total of 48 pens). Triplets are numbered from 1 to 16, systematically decreasing in odor-intensity from 1 (strongest) to 16 (weakest). For each triplet, one pen is impregnated with n-Butanol (BUT) while the other two only contain the solvent. The subject is blindfolded and required to identify the pen with odorant amongst the triplet. As part of the instructions, they are first presented with the most intense BUT-pen (Pen Nr. 1) in order to familiarize the subject with the smell of the target-pen. Following from here, in each trial, the participant tries three pens in a given triplet and decides which one is the odd one and then moves on to the next trial. The experimenter makes sure that there is roughly a 30-s interval between the beginning of consecutive trials.

The Threshold test follows a forced-choice staircase procedure. The individual vials of each triplet are presented in a varying cycle, which repeats until completion. Initially, the triplets 16, 14, 12, and so on are presented in descending order until the subject successfully identifies a triplet twice in a row. Should the subject fail to do so by the time Triplet No. 1 is reached, they are excluded from further testing due to suspected anosmia. Otherwise, the first triplet identified correctly twice in succession is documented as one turning point. Subsequently, the participant is continuously presented with the triplet with the next higher intensity twice, until they make an incorrect choice, which will then be marked as the next turning point. From that point, the next lower intensity triplet is presented until the subject again identifies it correctly twice in a row, at which point the order will be reversed once more. The test concludes when seven turning points have been recorded. The *T* score is defined as the mean of the last four turning points.

#### Discrimination test

Discrimination test also uses 16 triplets of pens. In each triplet, two pens are infused with the same odor (Non-Target), while the third pen contains a different odor (Target). The participant must discriminate the odd (Target) pen in each triplet. Here too, the participant is blindfolded. The participant goes through the 16 triplets only once. Timings are similar to the Threshold test. The D score is defined as the total number of correctly discriminated odd (Target) pens.

#### Identification test

The Identification test consists of 16 pens (no triplets), each emanating a unique smell. The participant is not blindfolded. The smell of each pen is matched to one of four multiple-choice words printed on a card associated with that pen. For individual testing of identification, both English and German multiple-choice card sets were available. Participants freely selected the version of their choice and completed the task using their preferred language. A previous study has shown that the information format, i.e., verbal or non-verbal information (photographs, drawings), does not influence the scores obtained.^58^ Once again, the interval between the presentation of each pen is around 30 s. The I score is defined as the total number of correctly identified odors.

Crucially, the Identification test uses a four-alternative forced-choice format (chance-level performance = 25%), whereas the Discrimination test is a three-alternative oddity task (chance-level performance = 33.3%). Relatedly, all comparative analyses reported are either model tasks explicitly or are reported separately by task to respect these baseline differences that are inherent to the task.

#### Experimental procedure

The study consisted of two sessions conducted on separate days (Figure 1B). On Day 1, participants completed olfactory tasks (Threshold, Discrimination, Identification) individually to establish baseline performance. On Day 2, participants returned to go through the Discrimination and Identification tasks once again. This time, we scheduled them in pairs and in each trial, they first decided individually and then collaboratively. On Day 2, we did not conduct the Threshold test. This decision was made because the threshold test’s staircase paradigm did not allow for joint decision. In the staircase, the stimulus that a given participant receives in any trial depends on this history of their own personal mistakes. As a consequence, as soon as two participants disagree, the history of their subsequent stimuli will diverge, rendering joint decision-making meaningless. There was, on average, a 40.6 days (SD = 23.3) time gap between the two testing days.

During joint testing on Day 2, dyads were seated side-by-side at a table with a cardboard divider between them to prevent direct visual contact (Figure 1D). The experimenter sat across the table. Each trial began with individual responses—blindfolded for Discrimination, unblinded for Identification—submitted privately via hand gestures (e.g., showing 2 fingers to indicate pen #2 as choice) for Discrimination or via written sheets for Identification. Participants then discussed their answers for 30–45 s to reach a joint decision, which was recorded by the experimenter. All Day 2 discussions were conducted in English. Order of presentation pens to dyad members were counterbalanced, and feedback was not given.

All discussions were audio recorded via headset microphones to capture verbal communications and quantitatively explore possible reasoning strategies.

#### Dyadic matching procedure

We hypothesized, based on prior research on joint perception^1^ that dyadic collective benefit would covary with similarity of their individual perceptual sensitivities. Therefore, it was important for us to test collective decision-making in dyads that demonstrated the full range of similarity of sensitivity from “one very good and one very bad” all the way to “both equally good”. To achieve this, we conducted the Day 1 tests to identify the individual’s sensitivity in the Discrimination and Identification independently. As planned in the pre-registration, we used the combined Discrimination and Identification (DI) scores from Day 1 to match half of our participants into dyads with similar sensitivity and the other half into dyads with differing sensitivity.

The matching procedure was as follows: first, we ranked the participants according to their D + I scores. Next, we classified them into three categories (Figure 1C). The two middle quartiles (Figure 1C, gray bars) were categorised as the participants with average ability. The top (Figure 3A, red bars) and the bottom (Figure 1C, blue bars) quartiles were categorised as high ability and low ability participants, respectively. Members of the average ability category were randomly matched with one another. These were our high similarity dyads (mean D + I score difference: 1.50 points, SD = 1.27, Min = 0, Max = 3). For low similarity dyads, randomly selected members of the high ability category were paired with randomly selected members of the low ability group (mean difference in D + I score 6.00 points, SD = 2.45, Min = 4, Max = 12).

#### Audio recordings

Participants’ verbal exchange during the collective phase was recorded via the “Multi-Plattform-Stereo-Headset PC 5 CHAT EPOS”. Each participant had a unique microphone for themselves. Voice recordings were started by the experimenter at the beginning of the sessions. Recordings were saved via “Audacity” (version 3.5.1) application, with a sampling rate of 16k Hz over 2 channels, to a local computer. Recorded audio files merged into a single channel and transcribed into text by Restream. Transcriptions were manually checked by 2 experimenters independently. Then, conversations were again manually labeled based on task and trial number by the experimenters. Corrected transcripts were parsed with “python-docx” to label each utterance with its experimental phase, trial number, and speaker. A lightweight routine tokenized every utterance by whitespace and, for each trial, aggregated (i) words and turns per speaker and (ii) a flag identifying which participant spoke first. The resulting per-trial table was exported for further statistical analysis. Finally, transcribed texts were manually inspected for every word & phrase that includes common confidence, certainty, and uncertainty expressions (e.g., “I’m sure”, “I’m not sure”, “I’m confident”, “Maybe” etc.); which were counted and stored per participant and per trial. This rating procedure was done by 3 raters and inter-rater reliability reported in the supplemental information.

### Quantification and statistical analysis

Across all statistical tests, we report full diagnostics and, where relevant, sensitivity (minimum detectable effect; MDE) in the supplemental information (Section D-E). For t-tests (independent and paired), we assess normality of (group-wise or paired-difference) residuals and provide nonparametric robustness checks; sensitivity analyses were conducted for regression models only. For ordinary least-squares (OLS) regressions, we report residual normality, homoskedasticity checks, and influence diagnostics; sensitivity is summarized as the partial R^2^ detectable at α = 0.05 (two-sided) with 80% power, obtained from the noncentral-F formulation. For linear mixed models (Gaussian) and generalized mixed models (binomial and Poisson), we document convergence/singularity checks and DHARMa residual diagnostics (uniformity and dispersion, with zero-inflation where applicable); sensitivity is reported on the model’s natural scale using Wald approximations with the fitted standard errors and degrees of freedom—i.e., as an MDE difference on the outcome scale (or per +1 SD of the predictor) for LMMs, as odds ratio MDEs for logistic models (per +1 SD for continuous predictors), as rate-ratio MDEs for Poisson models, and, for intercept-only logistic models, as the smallest detectable departure from chance (with equivalent probability thresholds). Exact model formulas, model-specific MDE values, and all diagnostic outputs are provided under “Sensitivity Analyses” and “Model Specifications & Diagnostics” in the supplemental information.
