# Supplementary material for: Human pairs show collective benefit in olfactory perception despite individual differences and verbal limits
Source: iScience. 2025 Dec 24;29(1):114535. doi: 10.1016/j.isci.2025.114535 (PMC12818292; doi:10.1016/j.isci.2025.114535)
Supplement: Document S1. Figure S1 and Tables S1–S46 [file mmc1.pdf]

iScience, Volume *29*

Supplemental information

Human pairs show collective benefit in olfactory perception despite individual differences and verbal limits

Mustafa Yavuz, Saman Sayahpour, Bahador Bahrami, and Ophelia Deroy

Supplementary Material

A) Individual Test Scores

Table S1. Descriptive Statistics for Olfactory Test Scores (N = 53)

Measure	M	SD	Min	Max
Threshold Test (T)	11.13	2.79	5.50	15.75
Discrimination Test (D)	11.66	1.97	8.00	15.00
Identification Test (I)	11.62	1.75	8.00	15.00
Discrimination + Identification (DI)	23.28	2.78	16.00	29.00
Total Score (DTI)	34.41	4.61	22.50	43.50

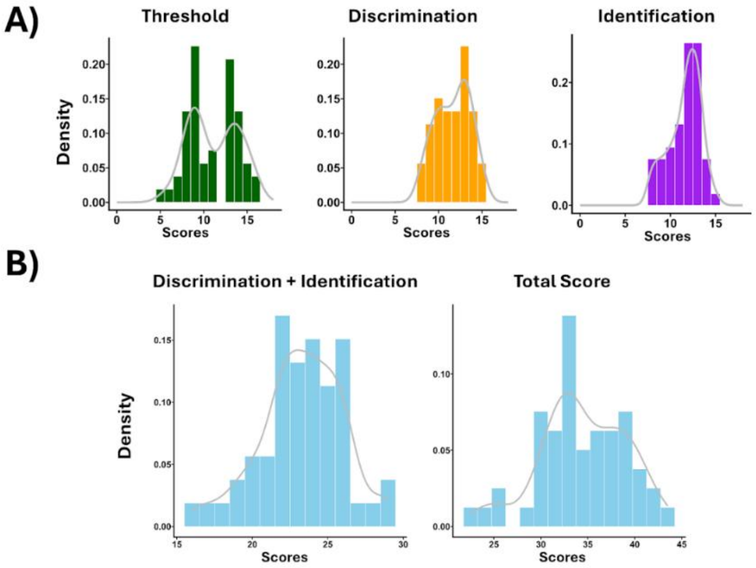

Figure S1. Distribution of individual scores across subtests and total scores.
Panel A shows distribution of individuals scores for threshold, discrimination and identification subtests (from left to right). Panel B shows, from left to right, the total scores for the sum of discrimination and identification subtests and total cumulative score, respectively.

B) Ability Balance Check

Table S2. Ability balance: Inferential tests and diagnostics

Task	Welch t(df)	p-value	95% CI of Mean Difference	Shapiro–Wilk (Similar)	p-value	Shapiro–Wilk (Different)	p-value	Wilcoxon W	p-value
Discrimination	−0.092 (18.00)	0.928	[−1.193, 1.093]	0.914	0.312	0.968	0.875	50	0.970
Identification	0.221 (17.62)	0.828	[−0.854, 1.054]	0.901	0.227	0.934	0.487	56	0.672

Table S3. Moderation models: coefficients, CIs, p, R^2, and HC3 p values per model

Task	Ability bands based on	CB denominator	Similarity term	β [95% CI]	p	Band (High) β [95% CI]	p	Interaction β [95% CI]	p	R^2	HC3 p (Sim)	HC3 p (Band)	HC3 p (Int)
Discrimination	Day-1	Day-1	Sim_D1	0.958 [0.495, 1.421]	0.000	0.209 [−0.423, 0.841]	0.494	−0.515 [−1.263, 0.233]	0.164	0.747	0.011	0.525	0.238
Discrimination	Day-1	Day-2	Sim_D2	0.442 [0.020, 0.864]	0.041	−0.070 [−0.606, 0.467]	0.786	−0.081 [−0.724, 0.563]	0.794	0.509	0.030	0.750	0.775
Identification	Day-1	Day-1	Sim_D1	0.182 [−0.573, 0.937]	0.616	0.071 [−0.917, 1.060]	0.880	−0.113 [−1.275, 1.050]	0.840	0.018	0.797	0.915	0.891
Identification	Day-1	Day-2	Sim_D2	0.591 [−0.030, 1.212]	0.061	0.077 [−0.636, 0.790]	0.821	−0.070 [−0.870, 0.730]	0.855	0.373	0.364	0.906	0.919
Discrimination	Day-2	Day-1	Sim_D1	0.361 [−0.440, 1.161]	0.354	−1.002 [−2.307, 0.302]	0.123	1.168 [−0.329, 2.666]	0.118	0.379	0.278	0.181	0.190
Discrimination	Day-2	Day-2	Sim_D2	0.619 [0.239, 0.999]	0.003	0.208 [−0.697, 1.113]	0.633	−0.400 [−1.440, 0.639]	0.426	0.495	0.010	0.692	0.512
Identification	Day-2	Day-1	Sim_D1	0.004 [−0.498, 0.506]	0.987	0.222 [−0.305, 0.750]	0.385	−0.049 [−0.686, 0.589]	0.874	0.573	0.996	0.773	0.958
Identification	Day-2	Day-2	Sim_D2	0.559 [0.058, 1.060]	0.031	0.071 [−0.580, 0.721]	0.821	−0.079 [−0.820, 0.662]	0.824	0.368	0.130	0.873	0.873

Table S4. Diagnostics: normality, heteroskedasticity, influence/LOO, and rank correlation

Task	Bands	CB denominator	Shapiro–Wilk W (p)	Breusch–Pagan χ^2 (p)	Max Cook's D (row)	LOO Sim β [95% CI], p	R^2 (LOO)	Spearman ρ (p)
Discrimination	Day-1	Day-1	0.965 (0.639)	2.447 (0.118)	0.474 (11)	1.185 [0.735, 1.635], 0.000	0.814	0.457 (0.043)
Discrimination	Day-1	Day-2	0.918 (0.090)	1.506 (0.220)	0.247 (11)	0.338 [−0.040, 0.717], 0.076	0.468	0.366 (0.112)
Identification	Day-1	Day-1	0.962 (0.594)	0.307 (0.579)	1.031 (9)	−0.468 [−1.411, 0.474], 0.306	0.087	0.033 (0.892)
Identification	Day-1	Day-2	0.928 (0.142)	0.678 (0.410)	0.739 (13)	1.000 [0.457, 1.542], 0.001	0.614	0.616 (0.004)
Discrimination	Day-2	Day-1	0.985 (0.981)	4.170 (0.041)	0.348 (8)	0.361 [−0.363, 1.084], 0.305	0.236	0.457 (0.043)
Discrimination	Day-2	Day-2	0.955 (0.447)	0.810 (0.368)	0.290 (12)	0.619 [0.241, 0.997], 0.003	0.521	0.366 (0.112)
Identification	Day-2	Day-1	0.907 (0.057)	0.214 (0.644)	3.445 (9)	−0.775 [−1.663, 0.112], 0.082	0.605	0.033 (0.892)
Identification	Day-2	Day-2	0.951 (0.389)	0.297 (0.586)	0.350 (9)	0.363 [−0.161, 0.887], 0.160	0.356	0.616 (0.004)

Notes: "Bands" column indicates whether ability bands were formed from Day-1 or Day-2 individual means. HC3 p-values are shown per fixed effect in Table 1 to mirror the robust-SE checks used elsewhere. LOO rows report the Similarity term after removing the single highest Cook's D case and the resulting R^2(LOO).

C) Task Comparison of Collective Benefit

Table S5. Task comparison of collective benefit: Inferential test and diagnostics

Contrast (paired)	Paired t(df)	p-value	95% CI of Mean Difference	Shapiro–Wilk W	p-value	Wilcoxon V	p-value
Identification − Discrimination	−0.048 (19)	0.962	[−0.078, 0.074]	0.965	0.642	87	0.948

E) Statistical Model Specifications & Diagnostics

1) Individual performance and demographics (Age & Gender)

Table S6. Age effects (Day-1 subtests) with diagnostics

Task	Predictor	β	SE	t	p	Shapiro–Wilk W	Shapiro p	Breusch–Pagan χ²	BP p	Max Cook's D
Threshold	Age	0.162	0.093	1.738	0.088	0.939	0.010	0.004	0.949	0.387
Discrimination	Age	0.016	0.068	0.231	0.818	0.951	0.031	0.058	0.810	0.262
Identification	Age	0.162	0.056	2.908	0.005	0.969	0.180	2.971	0.085	0.156

Table S7. Gender effects (Day-1 subtests) with diagnostics

Task	Predictor (M vs F)	β	SE	t	p	Shapiro–Wilk W	Shapiro p	Breusch–Pagan χ²	BP p	Max Cook's D
Threshold	Gender	−0.093	0.844	−0.110	0.913	0.936	0.007	0.674	0.412	0.104
Discrimination	Gender	−0.588	0.590	−0.997	0.323	0.970	0.192	0.002	0.965	0.129
Identification	Gender	−0.265	0.526	−0.504	0.616	0.943	0.014	0.770	0.380	0.136

2) Trial-level metacognition

Models (binomial GLMM)

Discrimination: Accuracy ~ Confidence + (Confidence | Subject)

Identification: Accuracy ~ Confidence + (Confidence | Subject)

Table S8. Trial-level metacognition: fixed effects (GLMM)

Task	β (Confidence)	SE	z	p	OR	95% CI (OR)
Discrimination	0.527	0.066	8.006	0.000	1.694	[1.489, 1.928]
Identification	0.695	0.077	9.030	0.000	2.004	[1.723, 2.330]

Table S9. GLMM diagnostics

Task	Singularity	Pearson φ	DHARMa uniformity (KS D)	p	DHARMa dispersion (ratio)	p
Discrimination	-	0.935	0.028	0.499	1.013	0.758
Identification	-	0.917	0.030	0.444	1.024	0.608

3) Dyad-level contrasts: collaborative vs individual benchmarks (Day-1)

Table S10. Dyad-level Day-1 benchmarks: paired contrasts and diagnostics

Contrast	Paired t (df)	p	95% CI of mean difference	Shapiro–Wilk W	p	Wilcoxon V	p
Discrimination: Dyad vs Best	1.291 (19)	0.212	[−0.342, 1.442]	0.915	0.081	111	0.255
Identification: Dyad vs Best	0.000 (19)	1.000	[−0.696, 0.696]	0.917	0.086	40	0.968
Discrimination: Dyad vs Average	4.528 (19)	0.000	[0.874, 2.376]	0.984	0.972	161	0.001
Identification: Dyad vs Average	3.101 (19)	0.006	[0.358, 1.842]	0.923	0.114	148	0.006

Notes: Shapiro–Wilk is computed on paired differences; Wilcoxon is the paired signed-rank test (no continuity correction).

4) Similarity and collective benefit (day-matched)

Models (OLS)
For each task × day: CB_Task_Day ~ Sim_Task_Day

Table S11. OLS results: CB ~ Similarity (day-matched)

Task (Day)	β (Similarity)	95% CI	t(18)	p	R^2
Discrimination (Day-1)	0.711	[0.136, 1.285]	2.599	0.018	0.273
Discrimination (Day-2)	0.343	[−0.043, 0.728]	1.868	0.078	0.162
Identification (Day-1)	0.079	[−0.357, 0.514]	0.381	0.708	0.008
Identification (Day-2)	0.524	[0.183, 0.865]	3.224	0.005	0.366

Table S12. Diagnostics for CB ~ Similarity models

Task (Day)	Shapiro–Wilk W	p	Breusch–Pagan χ^2	p	Max Cook’s D	LOO β	95% CI	p	R^2 (LOO)	HC3 t	p	Spearman ρ	p
Discrimination (Day-1)	0.979	0.916	2.871	0.090	0.474	0.496	[−0.020, 1.013]	0.058	0.195	2.331	0.032	0.457	0.043
Discrimination (Day-2)	0.941	0.254	0.927	0.336	0.225	0.282	[−0.041, 0.605]	0.083	0.166	1.853	0.080	0.366	0.112
Identification (Day-1)	0.953	0.421	0.197	0.657	0.891	−0.176	[−0.657, 0.305]	0.450	0.034	0.230	0.820	0.033	0.892
Identification (Day-2)	0.940	0.238	0.346	0.556	0.227	0.592	[0.289, 0.894]	0.001	0.500	2.988	0.008	0.616	0.004

Notes: LOO = leave-one-out refit after removing the highest Cook’s D case when leverage was notable; HC3 = heteroskedasticity-consistent (type HC3) t-test on the similarity slope; Spearman reports the rank correlation between CB and Similarity.

5) Ability similarity, conflict frequency, and conflict resolution

Models.

Paired task contrasts: conflict counts and conflict-resolution rates (Identification − Discrimination). OLS regressions:

Discrimination conflict count: Real_Conflict_Disc_Count ~ DISC_IND_Ratio_First
Identification conflict count: Real_Conflict_Ident_Count ~ IDF_IND_Ratio_First
Discrimination resolution rate: Real_Conflict_Disc_SuccessRate ~ DISC_IND_Ratio_First
Identification resolution rate: Real_Conflict_Ident_SuccessRate ~ IDF_IND_Ratio_First

Table S13. Paired task contrasts (Identification − Discrimination)

Contrast	Paired t(df)	p	95% CI of Mean Difference	Shapiro–Wilk W	p	Wilcoxon V	p
Conflict count	0.398 (19)	0.695	[−1.064, 1.564]	0.946	0.304	71	0.876
Resolution rate	1.636 (19)	0.118	[−0.023, 0.187]	0.943	0.276	134	0.112

Table S14. Ability similarity predicting conflict frequency and resolution

Model	Slope β	95% CI	t	p	R^2	Shapiro–Wilk W	p	Breusch–Pagan χ^2	p	Max Cook's D (row)	LOO β	95% CI (LOO)	t (LOO)	p (LOO)	R^2 (LOO)	HC3 t	p (HC3)	Spearman ρ	p
**Conflict count (Discrimination)**	−7.844	[−15.809, 0.121]	−2.069	0.053	0.192	0.936	0.204	1.077	0.299	0.541 (11)	−11.020	[−17.935, −4.105]	−3.362	0.004	0.399	−1.757	0.096	−0.501	0.024
**Conflict count (Identification)**	−6.674	[−12.522, −0.827]	−2.398	0.028	0.242	0.935	0.189	3.400	0.065	0.627 (5)	−9.189	[−14.047, −4.332]	−3.991	0.001	0.484	−1.978	0.063	−0.452	0.045
**Resolution rate (Discrimination)**	0.023	[−0.787, 0.833]	0.060	0.953	0.000	0.934	0.183	0.625	0.429	0.163 (12)	—	—	—	—	—	0.079	0.938	0.019	0.935
**Resolution rate (Identification)**	−0.087	[−0.918, 0.743]	−0.221	0.827	0.003	0.954	0.435	0.043	0.836	0.286 (6)	−0.329	[−1.142, 0.485]	−0.852	0.406	0.041	−0.206	0.839	−0.068	0.776

6) Verbal communication during dyadic decisions

Model (Gaussian LMM)

total_words ~ conflict * task + (1 | dyad); REML

Table S15. Task-Conflict type descriptives for total number of words

Task	n (no-conflict)	Mean (no-conflict)	SD (no-conflict)	n (conflict)	Mean (conflict)	SD (conflict)
Discrimination	210	27.776	28.428	110	77.991	40.888
Identification	216	17.769	21.167	104	58.471	27.622

Table S16. Fixed and random effects with diagnostics and model fit

Effect	β	95% CI	df	t	p	Singularity	DHARMa KS D	DHARMa KS p	Dispersion	Dispersion p
Intercept	28.598	[20.467, 36.729]	24.634	7.249	0.000	-	0.154	0.000	0.997	0.963
Conflict	47.823	[42.133, 53.513]	620.166	16.506	0.000	-	0.154	0.000	0.997	0.963

Table S16. Fixed and random effects with diagnostics and model fit

Effect	β	95% CI	df	t	p	Singularity	DHARMa KS D	DHARMa KS p	Dispersion	Dispersion p
Task	−11.104	[−15.728, −6.480]	617.942	−4.716	0.000	-	0.154	0.000	0.997	0.963
Interaction	−6.276	[−14.356, 1.803]	619.667	−1.525	0.128	-	0.154	0.000	0.997	0.963
Random effects (SD_dyad / σ_resid)			15.966 / 24.171							
Model fit (R^2 m/c; logLik; AIC; BIC; N trials; N dyads)			0.372 / 0.563; −2963.897; 5939.794; 5966.563; 640; 20							

7) Verbal communication and conflict-resolution success

Models (binomial GLMM)

Simple: Dyad_score ~ total_words + (1 | dyad)
Task-adjusted: Dyad_score ~ total_words + trial_type + (1 | dyad)
By task (simple within each task): Discrimination; Identification

Table S17. Conflict trials: GLMM fixed effects, diagnostics, and model fit

Model	Predictor	β	SE	z	p	OR	95% CI (OR)	Singularity	Overdisp (Pearson)	DHARMa KS D (p)	DHARMa Disp (p)
**Task Adjusted**	total_words	−0.016	0.005	−3.187	0.001	0.984	[0.974, 0.994]	-	0.985	0.031 (0.986)	1.001 (0.977)
	Task: Identification (vs Discrimination)	−0.748	0.333	−2.247	0.025	0.473	[0.246, 0.909]	-	0.985	0.031 (0.986)	1.001 (0.977)
Model Fit	*AIC* = 256.494; *BIC* = 269.958; logLik = −124.247; N(trials) = 214; R^2(m/c) = 0.098 / 0.124; SD(dyad) = 0.315										
**Simple**	total_words	−0.013	0.005	−2.770	0.006	0.987	[0.978, 0.996]	-	0.974	0.031 (0.988)	1.005 (0.968)
Model Fit	*AIC* = 259.737; *BIC* = 269.835; logLik = −126.869; N(trials) = 214; R^2(m/c) = 0.060 / 0.083; SD(dyad) = 0.284										
**Discrimination**	total_words	−0.009	0.005	−1.731	0.083	0.991	[0.981, 1.001]	TRUE	1.026	0.087 (0.377)	1.006 (0.986)
Model Fit	*AIC* = 129.813; *BIC* = 137.915; logLik = −61.907; N(trials) = 110; R^2(m/c) = 0.040 / 0.040; SD(dyad) = 0.000										
**Identification**	total_words	−0.032	0.011	−3.067	0.002	0.968	[0.949, 0.988]	-	0.961	0.099 (0.264)	1.002 (0.992)

Table S17. Conflict trials: GLMM fixed effects, diagnostics, and model fit

Model	Predictor	β	SE	z	p	OR	95% CI (OR)	Singularity	Overdisp (Pearson)	DHARMa KS D (p)	DHARMa Disp (p)
Model Fit	*AIC* = 126.421; *BIC* = 134.354; logLik = −60.210; N(trials) = 104; R^2(m/c) = 0.184 / 0.234; SD(dyad) = 0.464										

p-*values for the two by-task slopes are BH-adjusted (m = 2): Discrimination 0.083; Identification 0.004.*

8) Talkativeness and probability of individual answer being chosen

Model (binomial GLMM):

TalkativeWins ~ 1 + (1 | dyad); 102 conflict trials from 20 dyads.

Table S18. “More talkative wins?” — Identification conflicts (GLMM)

Effect	β	SE	z	p-value	OR [95% CI]	Probability [%] [95% CI]	Singularity	Overdispersion (Pearson)	DHARMa KS D	KS p-value	DHARMa Dispersion	Dispersion p-value
Intercept	-0.645	0.247	-2.609	0.009	0.525 [0.323, 0.852]	34.4 [24.4, 46.0]	-	0.926	0.066	0.759	1.013	1.000
Model fit	N = 102 trials; dyads = 20; logLik = -65.925; AIC = 135.850; BIC = 141.100; SD(Dyad) = 0.479 R^2 (marginal/conditional) = 0.000 / 0.065											

Notes: Probability is the inverse-logit of the intercept (prevalence of “talkative wins”).

9) Confidence-lexicon usage by conflict status and task

Model (Poisson GLMM)

total_conf_words ~ conflict * task + (1 | dyad); 639 trials, 20 dyads.

Table S19. Fixed effects, diagnostics, and model fits

Model	Term	β (log)	z	p	OR [95% CI]	Singularity	Pearson ratio	DHARMa KS p	DHARMa Disp p
Poisson GLMM	Conflict	1.205	17.235	0.000	3.336 [2.909, 3.825]	-	1.624	0.001	0.364
	Task	−0.367	−4.309	0.000	0.693 [0.587, 0.819]				
	Interaction	0.161	1.509	0.131	1.175 [0.953, 1.448]				
	Model fit & RE				N=640; dyad=20; logLik=−1177.187; AIC=2364.373; BIC=2386.681; R^2(m/c)=0.427/0.577; SD(dyad)=0.373				
NB2 GLMM	Conflict	1.231	14.281	0.000	3.424 [2.892, 4.055]	*NA*	1.251	0.258	0.327
	Task	−0.358	−3.767	0.000	0.699 [0.580, 0.842]				
	Interaction	0.183	1.416	0.157	1.200 [0.932, 1.545]				
	Model fit & RE				N=640; dyad=20; logLik=−1158.034; AIC=2328.068; BIC=2354.837; R^2(m/c)=0.369/0.508; SD(dyad)=0.397				

Note. DHARMa indicates non-uniform residuals and mild overdispersion (Pearson ratio > 1.2). The NB2 model (fit due to overdispersion) yields the same inference pattern with acceptable DHARMa diagnostics.

10) Confidence-lexicon and conflict resolution

Models (binomial GLMM)

Simple: Dyad_score ~ total_conf_words + (1 | dyad)
Task + interaction: Dyad_score ~ total_conf_words * trial_type + (1 | dyad)

Table S20. Fixed effects, diagnostics, and model fits

Model	Term	β (log-odds)	z	p	OR [95% CI]	Singularity	Pearson ratio	DHARMa KS p	DHARMa Disp p
Simple	Confidence-lexicon	−0.158	−2.658	0.008	0.853 [0.759, 0.959]	-	0.969	0.830	0.958
	Model fit & RE				N=214; dyad=20; logLik=−127.368; AIC=260.736; BIC=270.834; R^2(m/c)=0.052/0.077; SD(dyad)=0.295				
Task-adjusted	Confidence-lexicon	−0.126	−1.737	0.082	0.882 [0.765, 1.016]	-	0.971	0.996	0.997
	Task	0.403	0.548	0.584	1.496 [0.354, 6.329]				
	Interaction	−0.214	−1.614	0.106	0.808 [0.623, 1.047]				
	Model fit & RE				N=214; dyad=20; logLik=−123.821; AIC=257.642; BIC=274.472; R^2(m/c)=0.096/0.131; SD(dyad)=0.369				

11) Trial-by-trial verbosity vs. stable talkativeness (Identification conflicts)

Models (binomial GLMM):

Primary: Dyad_score ~ words_within + words_between + trait_imbalance + (1 | dyad)

Sensitivity (ALL-Identification traits): Dyad_score ~ words_within_all + words_between_all + trait_imbalance_all + (1 | dyad)

Table S21. Fixed effects, diagnostics, and model fits

Model	Predictor	β (SE)	z	p-value	OR [95% CI]	Singularity	Pearson ratio	DHARMa KS p	DHARMa Disp p
Primary	words_within	−0.044 (0.012)	−3.579	0.000	0.957 [0.934, 0.980]	-	1.024	0.457	0.987
	words_between	−0.011 (0.014)	−0.830	0.406	0.989 [0.963, 1.015]				
	trait_imbalance	−1.843 (2.292)	−0.804	0.421	0.158 [0.002, 14.146]				
	Model fit & RE				N = 104; dyad = 20; logLik = −58.186; AIC = 126.371; BIC = 139.593; R^2(m/c) = 0.217 / 0.234; SD(dyad) = 0.268				
Sensitivity	words_within_all	−0.038 (0.011)	−3.330	0.001	0.963 [0.941, 0.984]	-	1.012	0.390	0.979
	words_between_all	−0.012 (0.019)	−0.637	0.524	0.988 [0.951, 1.026]				
	trait_imbalance_all	−1.809 (2.197)	−0.824	0.410	0.164 [0.002, 12.144]				
	Model fit & RE				N = 104; dyad = 20; logLik = −59.011; AIC = 128.023; BIC = 141.245; R^2(m/c) = 0.201 / 0.237; SD(dyad) = 0.394				

D) Sensitivity Analyses

1) Day-1 ability as a function of Age and Gender (OLS)

Design & models: Six simple OLS regressions (df_2 = 51 each) predicting Day-1 subtests from Age (continuous) or Gender (M vs F). Sample: n = 53.

Sensitivity: At α = .05 (two-sided) and 80% power, the MDE for a single predictor is partial R^2 = 0.133.

Table S22. Day-1 ability ~ Age / Gender (OLS; observed R^2 vs. MDE)

Outcome	Predictor	Observed R^2	MDE (partial R^2)	Relative to MDE
Threshold	Age	0.056	0.133	Below MDE
Discrimination	Age	0.001	0.133	Below MDE
Identification	Age	0.142	0.133	Above MDE
Threshold	Gender	0.000	0.133	Below MDE

Table S22. Day-1 ability ~ Age / Gender (OLS; observed R^2 vs. MDE)

Outcome	Predictor	Observed R^2	MDE (partial R^2)	Relative to MDE
Discrimination	Gender	0.019	0.133	Below MDE
Identification	Gender	0.005	0.133	Below MDE

2) Similarity and collective benefit (day-matched) OLSs

Table S23. Similarity and collective benefit (day-matched): observed R^2 vs. MDE

Task	Similarity	Observed R^2	MDE (partial R^2)	Relative to MDE
Discrimination	Day-1	0.273	0.305	Below MDE
Discrimination	Day-2	0.162	0.305	Below MDE
Identification	Day-1	0.008	0.305	Below MDE
Identification	Day-2	0.366	0.305	Above MDE

3) Ability similarity, conflict frequency, and conflict resolution OLSs

Table S24. Ability similarity predicting conflict outcomes (OLS): observed R^2 vs. MDE

Outcome	Task	Observed R^2	MDE (partial R^2)	Relative to MDE
Conflict count	Discrimination	0.192	0.305	Below MDE
Conflict count	Identification	0.242	0.305	Below MDE
Resolution rate	Discrimination	0.000	0.305	Below MDE
Resolution rate	Identification	0.003	0.305	Below MDE

4) Verbal communication during dyadic decisions

Table S25. Verbal communication during joint decisions (LMM): observed effects vs. MDE

Effect	Observed $\lvert\beta\rvert$ (words)	MDE (words)	Relative to MDE
Conflict	47.823	8.130	Above MDE
Task	11.104	6.607	Above MDE
Interaction	6.276	11.544	Below MDE

5) Verbal communication and conflict-resolution success (GLMMs)

Table S25. Verbal communication and conflict resolution (GLMM): observed effects vs. MDE

Model	β (per word)	Observed log-odds difference	Observed OR	MDE (log-odds)	MDE (OR)	Relative to MDE
Simple	−0.0128	−0.464	0.629	0.469	1.599	Below MDE
Task-adjusted	−0.0162	−0.587	0.556	0.516	1.676	Exceeds MDE
Discrimination only	−0.0090	−0.369	0.692	0.597	1.816	Below MDE
Identification only	−0.0322	−0.890	0.411	0.813	2.254	Exceeds MDE

6) Talkativeness and probability of individual answer being chosen (GLMM)

Table S26. Talkativeness and probability of individual answer being chosen (GLMM): observed effects vs. MDE

Model	Observed log-odds	Observed OR	Observed probability	MDE (log-odds)	MDE (OR)	Probability thresholds	Relative to MDE
Intercept-only (talkative wins)	−0.645	0.525	34.4%	0.693	2.000	≥ 66.7% or ≤ 33.3%	Below MDE

Note. Observed probability 34.4% (95% CI [24.4%, 46.0%]).

7) Confidence-lexicon usage by conflict status and task (Poisson GLMM)

Table S27. Confidence-lexicon usage by conflict and task (Poisson GLMM): Observed effects vs. MDE

Effect	Observed (log)	Observed (RR)	MDE (log)	MDE (RR)	Relative to MDE
Conflict (conflict vs no-conflict)	1.205	3.336	0.196	1.216	Exceeds MDE
Task (Identification vs Discrimination)	−0.367	0.693	0.238	1.269	Exceeds MDE
Interaction	0.161	1.175	0.299	1.349	Below MDE

8) Conflict trials: dyad success as a function of confidence-lexicon counts (GLMM)

Table S28. Dyad success by confidence-lexicon (GLMM): Observed effects vs. MDE

Model / Effect	Observed (log-odds)	Observed (OR)	MDE (log-odds)	MDE (OR)	Relative to MDE
Simple	−0.431	0.65	0.454	1.58	Below MDE
Task-adjusted — confidence	−0.343	0.71	0.553	1.74	Below MDE
Task-adjusted — interaction	−0.583	0.56	1.008	2.74	Below MDE

Table S28. Dyad success by confidence-lexicon (GLMM): Observed effects vs. MDE

Model / Effect	Observed (log-odds)	Observed (OR)	MDE (log-odds)	MDE (OR)	Relative to MDE
Task-adjusted — task main effect	0.403	1.50	2.061	7.86	Below MDE

9) Identification conflicts: within-/between-dyad talkativeness and trait imbalance (GLMMs)

Table S29. Identification conflicts: within-/between-dyad talkativeness and trait imbalance (GLMMs): Observed effects vs. MDE

Model (trait source)	Predictor	SD used	MDE (log-odds)	MDE (OR)	Observed log-odds per SD	Observed OR per SD	Relative to MDE
Conflict-only	Within-dyad deviation	21.119	0.729	2.074	−0.929	0.395	**Above MDE**
	Between-dyad mean	17.804	0.674	1.962	−0.199	0.820	Below MDE
	Trait imbalance	0.102	0.654	1.924	−0.188	0.829	Below MDE
All-Identification	Within-dyad deviation (all-ident mean)	23.176	0.743	2.102	−0.883	0.414	Above MDE
	Between-dyad mean (all-ident mean)	13.221	0.711	2.035	−0.161	0.851	Below MDE
	Trait imbalance (all-ident mean)	0.111	0.681	1.975	−0.201	0.818	Below MDE

F) Confidence-Word Annotation: Coders, Blinding, Reliability, and Agreement

Table S30. Intraclass correlation (ICC) and Krippendorff's alpha

Metric	Interpretation target	Estimate	95% CI	95% CI
ICC (2,1) (single-measure)	Reliability of one randomly chosen coder	0.822	0.738	0.874
ICC (2,3) (average-measure)	Reliability of the mean of three coders	0.933	0.894	0.954
Krippendorff's α (interval)	Overall agreement for interval-scaled counts	0.820	—	—

Notes. ICC(2,X) denotes two-way random effects with absolute agreement; ICC(2,1) is the single measure coefficient, and ICC(2,3) pertains to the average of three raters.

Table S31. Pairwise concordance (Lin's CCC)

Rater pair	CCC	95% CI lower	95% CI upper
Rater 1 – Rater 2	0.913	0.900	0.924
Rater 1 – Rater 3	0.806	0.783	0.828
Rater 2 – Rater 3	0.728	0.700	0.754

Table S32. Bland–Altman and tolerance (±1 count)

Rater pair	Mean difference	LoA lower	LoA upper	% within ±1
Rater 1 – Rater 2	−0.444	−2.922	2.035	81.6%
Rater 1 – Rater 3	+0.705	−2.222	3.632	79.4%
Rater 2 – Rater 3	+1.148	−2.514	4.811	70.9%

Robustness of results to raters

1) Confidence lexicon by conflict status and task type (Poisson GLMM)
Outcome variable was trial-level count of confidence words.

Model Specification:

$total\_conf\_words \sim conflict \times trial\_type + (1 \mid dyad)$

When the outcome was an average across raters, the mean was rounded to the nearest integer due to using Poisson.

Table S33. Count model (Poisson GLMM; outcome = number of confidence words)

**Effect**	**R1 (RR, p)**	**Avg 3 (RR, p)**	**Avg 2–3 (RR, p)**	**R2 (RR, p)**	**R3 (RR, p)**
Conflict (main)	3.280 (p<0.001)	3.336 (p<0.001)	3.262 (p<0.001)	3.032 (p<0.001)	3.847 (p<0.001)
Task: Identification (main)	0.670 (p<0.001)	0.693 (p<0.001)	0.683 (p<0.001)	0.658 (p<0.001)	0.764 (p<0.001)
Conflict × Task	1.178 (p=0.117)	1.175 (p=0.131)	1.227 (p=0.057)	***1.225 (p=0.035)***	1.113 (p=0.388)

2) Dyadic accuracy in conflict trials by confidence lexicon (logistic GLMM).

Model Specifications:

a) Simple Model:

Dyad_score ~ conf_words + (1 | dyad)

Table S34. Simple model

**Effect**	**R1 (OR, p)**	**Avg 3 (OR, p)**	**Avg 2–3 (OR, p)**	**R2 (OR, p)**	**R3 (OR, p)**
Confidence words	0.894 (p=0.0266)	0.853 (p=0.0079)	0.838 (p=0.0045)	0.864 (p=0.0030)	0.849 (p=0.0292)

OR = odds ratio. “Avg 3” = mean of all three raters; “Avg 2–3” = mean of the two blind raters (Raters 2 and 3).

b) Full Model:

Dyad_score ~ conf_words × trial_type + (1 | dyad)

When coders’ averages were used as predictors, the unrounded mean was entered as a continuous variable.

Table S35. Full model

Effect	R1 (OR, p)	Avg 3 (OR, p)	Avg 2–3 (OR, p)	R2 (OR, p)	R3 (OR, p)
Confidence words (main)	0.898 (p=0.0816)	0.882 (p=0.0824)	0.880 (p=0.0951)	0.904 (p=0.0878)	0.870 (p=0.157)
Task: Identification (main)	0.914 (p=0.891)	1.496 (p=0.584)	1.856 (p=0.416)	1.933 (p=0.354)	1.050 (p=0.947)
Confidence × Task	0.902 (p=0.357)	0.808 (p=0.106)	0.771 (p=0.0608)	***0.794 (p=0.0327)***	0.859 (p=0.356)

Notes. RR = rate ratio; OR = odds ratio. “Avg 3” = mean of all three raters; “Avg 2–3” = mean of the two blind raters (Raters 2 and 3).

G) Native Language Status: Composition and effects on performance and speech metrics

Table S36. Native languages of the sample (Day 1 & Day 2)

Language	Day 1 N	Day 1 %	Day 2 N	Day 2 %
German	21	39.6	18	45.0
English	4	7.5	3	7.5
Chinese (Mandarin)	4	7.5	2	5.0
Turkish	4	7.5	3	7.5
Spanish	3	5.7	3	7.5
Arabic	2	3.8	1	2.5
Indonesian	2	3.8	1	2.5
Miscellaneous	2	3.8	2	5.0
Portuguese	2	3.8	2	5.0
Russian	2	3.8	1	2.5
Azerbaijani	1	1.9	1	2.5
Bulgarian	1	1.9	1	2.5
Danish	1	1.9	1	2.5
Hindi	1	1.9	1	2.5
Not stated	1	1.9	0	0.0

Table S36. Native languages of the sample (Day 1 & Day 2)

Language	Day 1 N	Day 1 %	Day 2 N	Day 2 %
Polish	1	1.9	0	0.0
Ukrainian	1	1.9	0	0.0

1) Individual task performance by native-English status

Table S37. Day 1 (N = 53; 4 native English) performance by native language

**Task**	**β**	**SE**	**t(df)**	**p**
Discrimination	0.097	1.035	0.09 (51)	0.93
Identification	−0.133	0.916	−0.15 (51)	0.89

Table S38. Day 2 (N = 40; 3 native English) performance by native language

**Task**	**β**	**SE**	**t(df)**	**p**
Individual Discrimination	1.333	1.227	1.09 (38)	0.28
Individual Identification	−0.225	0.965	−0.23 (38)	0.82

2) Speech metrics by native-English status (dyadic session)

Total words: mean_words ~ Lang_ENG × task + (1 | person)

Table S39. Total words by native language

**Fixed effect**	**β**	**SE**	**t(df)**	**p**
Lang_ENG (ENG vs Other)	4.304	6.507	0.661 (58.7)	0.51
Task (Identification vs Discrimination)	−6.532	1.704	−3.834 (38)	0.00046
Interaction (Lang_ENG × Task)	−6.510	6.221	−1.046 (38)	0.302

Confidence-lexicon words: mean_conf ~ Lang_ENG × task + (1 | person)

Table S40. Confidence lexicon by native language

Fixed effect	β	SE	t(df)	p
Lang_ENG (ENG vs Other)	0.202	0.492	0.411 (61.0)	0.68
Task (Identification vs Discrimination)	−0.402	0.135	−2.970 (38)	0.005
Interaction (Lang_ENG × Task)	−0.536	0.494	−1.083 (38)	0.286

3) Individual task performance by English/German native-language status

Table S41. Confidence lexicon by native language

Task	β	SE	t(df)	p
Discrimination	−0.039	0.547	−0.07 (51)	0.94
Identification	0.411	0.482	0.85 (51)	0.40

Table S42. Day 2 performance by native language (English&German)

Task	β	SE	t(df)	p
Individual Discrimination	0.191	0.656	0.29 (38)	0.77
Individual Identification	−0.038	0.509	−0.07 (38)	0.94

4) Speech metrics by English/German native-language status (dyadic session)

Total words: mean_words ~ ENG/DE × task + (1 | person)

Table S43. Total words by native language (English&German)

Fixed effect	β	SE	t(df)	p
ENG/DE (ENG/DE vs Other)	−2.825	3.413	−0.83 (59.54)	0.41
Task (Identification vs Discrimination)	−7.166	2.350	−3.05 (38)	0.004
Interaction (ENG/DE × Task)	0.291	3.324	0.09 (38)	0.93

Confidence-lexicon words: mean_conf ~ ENG/DE × task + (1 | person)

Table S44. Confidence lexicon by native language (English&German)

Fixed effect	β	SE	t(df)	p
ENG/DE (ENG/DE vs Other)	−0.153	0.259	−0.59 (61.87)	0.56
Task (Identification vs Discrimination)	−0.413	0.187	−2.21 (38)	0.033
Interaction (ENG/DE × Task)	−0.059	0.264	−0.23 (38)	0.82

H) Pre-discussion states and dyadic performance

Table S45. Dyadic accuracy by pre-discussion states and task type

Task	Pre-discussion state	Trials (n)	Dyad correct (n)	Proportion correct (%)	95% CI (%)
Discrimination	Both correct	185	185	100.0	[98.0, 100.0]
Discrimination	A correct	52	38	73.1	[59.7, 83.2]
Discrimination	B correct	57	42	73.7	[61.0, 83.4]
Discrimination	Both wrong	25	1	4.0	[0.7, 19.5]
Identification	Both correct	185	185	100.0	[98.0, 100.0]
Identification	A correct	48	34	70.8	[56.8, 81.8]
Identification	B correct	56	34	60.7	[47.6, 72.4]
Identification	Both wrong	30	0	0.0	[0.0, 11.4]

Model specification
We fit a mixed-effects logistic regression with a random intercept for dyad:

dyad_score ~ pre_state × task + (1 | dyad)

Table S46. GLMM of dyadic accuracy in one-correct trials (A_only/B_only) with task as moderator

Predictor	β (log-odds)	SE	95% CI (log-odds)	OR	95% CI (OR)
Intercept (A_only, Discrimination)	1.000	0.320	[0.375, 1.630]	2.72	[1.46, 5.10]
B_only (vs A_only)	0.065	0.440	[-0.797, 0.927]	1.07	[0.45, 2.53]
Identification (vs Discrimination)	-0.113	0.449	[-0.994, 0.768]	0.89	[0.37, 2.16]

Table S46. GLMM of dyadic accuracy in one-correct trials (A_only/B_only) with task as moderator

Predictor	β (log-odds)	SE	95% CI (log-odds)	OR	95% CI (OR)
B_only × Identification	-0.509	0.609	[-1.700, 0.684]	0.60	[0.18, 1.98]
